# Differential association of hyperglucagonemia with C-peptide levels in diabetic ketosis/ketoacidosis and hyperosmolar hyperglycemic state

**DOI:** 10.1007/s13340-025-00852-8

**Published:** 2025-11-23

**Authors:** Tatsuya Iida, Sayuri Nii, Hiroto Nishikawa, Eriko Kodama, Hideyuki Imai, Mai Hashizume, Rie Tadokoro, Chiho Sugisawa, Toru Iizaka, Fumiko Otsuka, Shoichiro Nagasaka

**Affiliations:** Division of Diabetes, Metabolism, and Endocrinology, Showa Medical University Fujigaoka Hospital, 1-30 Fujigaoka, Aoba-ku, Yokohama, Kanagawa 227-8501 Japan

**Keywords:** Glucagon, Ketone bodies, C-peptide, Diabetic ketosis/ketoacidosis, Hyperosmolar hyperglycemic state

## Abstract

**Aims/introduction:**

Glucagon plays a pivotal role in the development of hyperglycemia in diabetes mellitus. The purpose of this study was to investigate the hypothesis that hyperglucagonemia based on measurements of pancreas-specific glucagon is present in diabetic ketosis/ketoacidosis (DK/DKA) and hyperosmolar hyperglycemic state (HHS), and if so, to explore factors contributing to that hyperglucagonemia.

**Materials and methods:**

A total of 109 patients (92 with DK/DKA, and 17 with HHS) were investigated. Pancreas-specific glucagon levels were measured with a sandwich enzyme-linked immunosorbent assay at treatment initiation. The relationships of plasma glucagon levels, serum ketone bodies levels, and endogenous insulin secretion were assessed. The change in plasma glucagon levels after treatment was also assessed.

**Results:**

The median plasma glucagon level was significantly higher in the HHS group (142.9 pg/mL) than in the DK/DKA group (63.6 pg/mL). In the DK/DKA group, the plasma glucagon level was positively correlated with the serum ketone bodies level (ρ = 0.55, *P* < 0.0001), but there was no correlation in the HHS group. In the DK/DKA group, a negative correlation was seen between the plasma glucagon level and the serum C-peptide immunoreactivity (CPR)/plasma glucose ratio in type 1 diabetes patients (n = 26) (ρ = − 0.67, *P* = 0.0002). In the HHS group, a positive correlation was seen between the plasma glucagon level and the serum CPR/plasma glucose ratio (ρ = 0.71, *P* = 0.0013). The plasma glucagon level was significantly lower after treatment in both the DK/DKA and HHS groups.

**Conclusions:**

Hyperglucagonemia was found in DK/DKA and HHS with pancreas-specific glucagon measurements. The results suggest that the causes of hyperglucagonemia differ in DK/DKA due to type 1 diabetes mellitus and HHS.

**Supplementary Information:**

The online version contains supplementary material available at 10.1007/s13340-025-00852-8.

## Introduction

The glucagon precursor proglucagon is expressed in pancreatic α cells and intestinal tract L cells. Proglucagon is processed and broken down into peptides such as glucagon and glicentin-related polypeptide (GRPP). Glucagon, GRPP, and other peptides are formed in pancreatic α cells. Glicentin, GRPP, oxyntomodulin, and other peptides are formed in intestinal tract L cells [1].

Glucagon has a wide range of physiological actions, including glucose metabolism in the liver, regulation of amino acid metabolism, inhibition of glucose transport in muscle, lipolysis and heat production in adipose tissue, appetite suppression in the brain, and inhibition of gastric emptying and intestinal peristalsis [2]. In the liver, though glycolysis and glycogen production are inhibited, glycogen breakdown and gluconeogenesis are facilitated, and glucose production in the liver is increased [3]. Glucagon acts as a counterregulatory hormone to hypoglycemia. In lipid metabolism, it facilitates β-oxidation and inhibits lipid production [4]. In amino acid metabolism, it is involved in the metabolic rate-limiting step and facilitates gluconeogenesis from amino acids [3].

Glucagon secretion is directly regulated by circulating glucose concentrations. Glucagon secretion is inhibited in hyperglycemia and facilitated in hypoglycemia [2, 5–7]. Hypoglycemia is the strongest stimulator of glucagon secretion. Insulin binds to α cell insulin receptors and directly inhibits glucagon secretion by opening ATP-sensitive K^+^ channels, hyperpolarizing the membrane potential, and inhibiting Ca^2+^ influx. Insulin also indirectly inhibits glucagon secretion by facilitating somatostatin secretion, and somatostatin inhibits glucagon secretion by decreasing cAMP [2, 8].

Blood levels of glucagon in diabetes mellitus have been investigated using radioimmunoassays (RIAs). Hyperglucagonemia, despite hyperglycemia both during fasting and after eating, has previously been reported in patients with type 2 diabetes mellitus [9, 10]. Other studies have reported that hyperglucagonemia is related to insulin resistance and fatty liver [11, 12]. Glucagon excess is thought to be related to the pathogenesis of diabetes mellitus [13]. Insufficient insulin action is thought to contribute to this glucagon excess, but the detailed mechanisms are not well understood. There are also reports of hyperglucagonemia during hyperglycemic crises. Suggested causes of hyperglucagonemia include decreased insulin action during diabetic ketosis (DK) and diabetic ketoacidosis (DKA) and stress from marked hyperglycemia and dehydration during a hyperosmolar hyperglycemic state (HHS) [14, 15].

With RIA, glucagon is measured using antibodies to the C-terminal, but this also measures peptides with structures analogous to glucagon at the same time, and the results have been demonstrated to be higher than the actual glucagon levels [16]. The accuracy of glucagon measurements has improved in recent years with the development of sandwich enzyme-linked immunosorbent assays (ELISAs). By using antibodies to the N- and C-terminals, sandwich ELISA differentiates between glucagon and analogous peptides that were measured with RIA, and more accurate measurements of glucagon levels have become possible. The correlation of sandwich ELISA to liquid chromatography mass spectrometry was found to be better than that of RIA [16].

New findings on plasma glucagon levels have increased after the development of sandwich ELISAs, but there have been no reports of the pancreas-specific glucagon level in hyperglycemic crises. The purpose of this study was to investigate the hypothesis that hyperglucagonemia based on sandwich ELISA is present in DK/DKA and HHS, and if so, to explore factors contributing to that hyperglucagonemia.

## Materials and methods

### Participants

Patients (n = 168) who had been examined at our hospital during the period from August 2019 to October 2024, and in whom a hyperglycemic crisis was suspected and blood tests related to hyperglycemic crisis were performed, were identified. They included 145 patients who were diagnosed with DK, DKA, or HHS. After excluding 34 patients who had undergone insulin therapy prior to those blood tests and 2 patients who had pancreatic diabetes, a total of 109 patients were included. The background leading to DK/DKA and HHS was new-onset diabetes mellitus (n = 74), discontinuation of diabetes treatment (n = 12), and other factors such as sick days (n = 23).

### Definition of hyperglycemic crisis and diagnosis of type 1 and type 2 diabetes mellitus

A hyperglycemic crisis was diagnosed using the presence of hyperglycemia (≥ 200 mg/dL) and (1) arterial blood gas pH < 7.3, anion gap > 12 mEq/L, (2) excess ketone bodies production (confirmation of urine ketone bodies positivity or high serum ketone bodies levels), and (3) serum osmolality ≥ 320 mOSM/kg. In patients who fulfilled (1) and (2) (regardless of (3)), the hyperglycemic crisis was diagnosed as DKA. Patients with only (2) were diagnosed with DK, and patients in whom (3) was fulfilled and (1) was not seen (regardless of (2)) were diagnosed with HHS [17]. With these criteria, all subjects could be classified into the three conditions of DKA, DK, and HHS.

The type of diabetes mellitus was diagnosed as type 1 (n = 26) based on the presence of islet-specific autoantibodies and/or endogenous insulin deficiency (fasting serum C-peptide immunoreactivity (CPR) < 0.6 ng/mL, or in the case of fulminant type 1 diabetes mellitus < 0.3), and the remainder as type 2 diabetes mellitus (n = 83).

### Laboratory analyses for hyperglycemic crisis

Laboratory tests were conducted as the routine assessment for hyperglycemic crisis, including hematological and biochemical tests, and arterial blood gas analysis, plus measurements of serum ketone bodies, serum CPR, and pancreas-specific glucagon levels. Pancreas-specific glucagon was measured at a commercial laboratory (BML, Inc., Tokyo, Japan) with sandwich ELISA (Mercodia Glucagon ELISA, Mercodia AB, Uppsala, Sweden) [18]. The normal reference value for the fasting glucagon level at BML, Inc. is 5.4–55.0 pg/mL (https://uwb01.bml.co.jp/kensa/search/detail/5504841).

After patients were hospitalized with a hyperglycemic crisis, they received infusion and insulin treatment. After food intake and other factors stabilized, fasting glucagon levels were retested in a majority of patients (n = 83, DK/DKA group n = 72, HHS group n = 11) (median 76 h after admission).

### Statistical analysis

The results are shown as median and interquartile range values. The patients were divided into two groups: (1) DK/DKA group (DK 55 patients + DKA 37 patients = 92 patients), and (2) HHS group (17 patients). Since DK and DKA are continuous pathological conditions, variables of DK and DKA are combined and analyzed simultaneously. In fact, serum ketone bodies levels were strongly associated with arterial blood pH, anion gap and bicarbonate in the DK/DKA group, and substantial overlap was seen between DK and DKA in the associations of serum ketone bodies levels with anion gap and bicarbonate in arterial blood (Supplementary Figure).

Comparisons between the groups were made using the Wilcoxon test and Pearson’s chi-squared test. The relationships between the plasma glucagon levels and serum ketone bodies were analyzed using Spearman’s rank correlation coefficient in the (1) DK/DKA group and (2) HHS group, respectively.

Next, the relationships between the plasma glucagon level and plasma glucose, serum CPR, the serum CPR/plasma glucose ratio as an indicator of insulin secretion relative to hyperglycemia, serum osmolality, and serum creatinine were analyzed in type 1 diabetes mellitus in the DK/DKA group, type 2 diabetes mellitus in the DK/DKA group, and in the HHS group, respectively, using Spearman’s rank correlation coefficient. These five factors were selected as factors with potential effects on the plasma glucagon level. The pathogenesis of DK/DKA is considered to be different between type 1 and type 2 diabetes mellitus; insulin deficiency in type 1 diabetes and relative insulin deficiency against insulin resistance in type 2 diabetes [19]. In fact, there was a substantial difference in endogenous insulin secretion between type 1 and type 2 diabetes in this study. Therefore, type 1 and type 2 diabetes were separately analyzed in the DK/DKA group.

Third, as other factors with possible effects on the plasma glucagon level, duration of hyperglycemic symptoms (from onset of hyperglycemic symptoms to the current admission) and duration of diabetes (from diabetes diagnosis to the current admission) were estimated. The relationships between the plasma glucagon level and duration of hyperglycemic symptoms or duration of diabetes were analyzed in type 1 and type 2 diabetes mellitus in the DK/DKA group, respectively, using Spearman’s rank correlation coefficient. In addition, the relationships between duration of hyperglycemic symptoms or duration of diabetes, and serum CPR and serum CPR/plasma glucose ratio were analyzed. The HHS group was excluded from this analysis, because duration of hyperglycemic symptoms could not be clearly determined. Finally, the plasma glucagon levels before and after treatment in the DK/DKA and HHS groups were analyzed with a paired *t*-test. Statistical analysis was performed using JMP software (JMP Pro 17, SAS Institute Inc., Cary, NC, USA).

## Results

### Clinical characteristics of the DK/DKA and HHS groups

The clinical characteristics of the two groups are shown in Table 1. The median age was significantly higher in the HHS group (85 years) than in the DK/DKA group (57 years; *P* < 0.0001). The DK/DKA group included 26 patients with type 1 diabetes mellitus and 66 with type 2 diabetes mellitus, whereas in the HHS group, all of the patients had type 2 diabetes mellitus. No significant difference in BMI was seen. The median plasma glucagon level was significantly higher in the HHS group (142.9 pg/mL) than in the DK/DKA group (63.6 pg/mL; *P* = 0.0041). The median plasma glucose level was also significantly higher in the HHS group (791 mg/dL) than in the DK/DKA group (484 mg/dL; *P* < 0.0001). The median serum ketone bodies level was significantly higher in the DK/DKA group (6224 μmol/L) than in the HHS group (1771 μmol/L; *P* = 0.0013). No significant differences were seen in arterial blood pH and the anion gap between the DK/DKA group and the HHS group, but in the DK/DKA group, arterial blood pH was lower and the anion gap was higher in patients with DKA. The median arterial bicarbonate level was 24.2 mEq/L in the HHS group and 18.5 mEq/L in the DK/DKA group (8.8 mEq/L with DKA), significantly higher in the HHS group (*P* = 0.0005). Serum creatinine and serum osmolality were both significantly higher in the HHS group. The median serum CPR was significantly higher in the HHS group (2.7 ng/mL) than in the DK/DKA group (1.4 ng/mL; 1.1 ng/mL with DKA; *P* = 0.001). No significant difference was seen in the serum CPR/plasma glucose ratio between the groups, but the median value was low in the DKA group (0.14).

**Table 1:** Clinical characteristics of the DK/DKA group and the HHS group

	DK/DKA group	HHS group	*P* value
Number	92 DK 55/DKA 37	17	
Age (years)	57 (41–68)	85 (80–90)	< 0.0001
Male/Female	61/31	5/12	0.0044
Type of diabetes mellitus (type 1/type 2)	26/66	0/17	0.011
BMI (kg/m^2^)	22.9 (20.1–26.8)	23.1 (19.0–24.3)	0.26
Plasma glucagon (pg/mL)	63.6 (34.0–131.6) DK 44.8/DKA 133.8	142.9 (76.1–283.95)	0.0041
HbA1c (%)	12.5 (10.3–14.4)	11.0 (8.5–12.1)	0.014
Plasma glucose (mg/dL)	484 (331–678)	791 (686–957)	< 0.0001
Serum ketone bodies (μmol/L)	6224 (1612–11,506)	1771 (334–3992)	0.0013
Arterial blood pH	7.37 (7.24–7.42) DK 7.41/DKA 7.19	7.41 (7.35–7.42)	0.07
Bicarbonate in arterial blood (mEq/L)	18.5 (10.3–22.2) DK 22.0/DKA 8.8	24.2 (19.3–27.4)	0.0005
Anion gap in arterial blood (mEq/L)	18.6 (14.2–25.4) DK 14.9 / DKA 26.0	17.7 (15.9–19.5)	0.44
Lactic acid in arterial blood (mmol/L)	1.6 (1.1–2.7)	2.2 (1.9–2.7)	0.049
Serum creatinine (mg/dL)	0.84 (0.63–1.31)	1.39 (1.01–1.66)	0.0058
Serum osmolality (mOSM)	306 (291–331)	368 (351–399)	< 0.0001
Serum C-peptide immunoreactivity (ng/mL)	1.4 (0.7–2.4) DK 1.7/DKA 1.1	2.7 (1.5–3.8)	0.001
C-peptide immunoreactivity/glucose	0.34 (0.15–0.57) DK 0.43/DKA 0.14	0.35 (0.18–0.66)	0.59

Median and interquartile ranges (in parenthesis) are shown

C-peptide immunoreactivity/glucose is shown as calculated values multiplied by 100

*BMI* body mass index, *DK* diabetic ketosis, *DKA* diabetic ketoacidosis, *HHS* hyperosmolar hyperglycemic state

### Clinical characteristics of type 1 and type 2 diabetes mellitus in the DK/DKA group

The characteristics of type 1 and type 2 diabetes mellitus in the DK/DKA group are shown in Table 2. BMI was significantly higher in patients with type 2 diabetes mellitus. No significant differences were seen in the median plasma glucagon level, but the median value was higher in patients with DKA in both groups. The median serum ketone bodies level was significantly higher, arterial blood pH and bicarbonate level were significantly lower in patients with type 1 diabetes mellitus. The median serum CPR and serum CPR/plasma glucose ratio was significantly lower in patients with type 1 diabetes mellitus, especially in those with DKA.

**Table 2:** Clinical characteristics of type 1 and type 2 diabetes mellitus in the DK/DKA group

	DK/DKA group		P value
	Type 1 diabetes	Type 2 diabetes	
Number	26 DK 13/DKA 13	66 DK 42/DKA 24	
Age (years)	62 (42–71)	53 (41–64)	0.27
Male/Female	14/12	47/19	0.14
BMI (kg/m^2^)	20.6 (17.6–22.5)	24.4 (21.1–28.1)	< 0.0001
Plasma glucagon (pg/mL)	59.0 (28.0–179.4) DK 32.9/DKA 178.8	66.5 (36.9–130.8) DK 48.3/DKA 132.1	0.67
HbA1c (%)	10.6 (7.8–14.6)	12.7 (10.9–14.3)	0.042
Plasma glucose (mg/dL)	555 (311–705)	460 (334–669)	0.71
Serum ketone bodies (μmol/L)	9824 (3864–14,726)	4793 (1410–14624)	0.015
Arterial blood pH	7.26 (7.15–7.39) DK 7.40/DKA 7.16	7.39 (7.28–7.43) DK 7.42/DKA 7.26	0.009
Bicarbonate in arterial blood (mEq/L)	11.9 (4.7–19.9) DK 20.0/DKA 6.4	19.9 (13.0–23.0) DK 22.0/DKA 10.2	0.013
Anion gap in arterial blood (mEq/L)	20.5 (16.4–29.3) DK 16.0 / DKA 28.8	18.3 (14.0–23.9) DK 14.7 / DKA 25.1	0.070
Lactic acid in arterial blood (mmol/L)	1.6 (1.1–3.0)	1.6 (1.2–2.3)	0.66
Serum creatinine (mg/dL)	0.86 (0.62–1.54)	0.84 (0.65–1.20)	0.96
Serum osmolality (mOSM)	308 (290–334)	304 (292–328)	0.93
Serum C-peptide immunoreactivity (ng/mL)	0.6 (0.1–1.2) DK 0.7/DKA 0.1	1.9 (1.3–2.7) DK 2.0/DKA 1.7	< 0.0001
C-peptide immunoreactivity/glucose	0.18 (0.01–0.32) DK 0.27/DKA 0.01	0.43 (0.25–0.62) DK 0.53/DKA 0.29	< 0.0001

Median and interquartile ranges (in parenthesis) are shown

C-peptide immunoreactivity/glucose is shown as calculated values multiplied by 100

*BMI* body mass index, *DK* diabetic ketosis, *DKA* diabetic ketoacidosis

### Associations of plasma glucagon levels with serum ketone bodies in the DK/DKA group and the HHS group

**Fig. 1:**
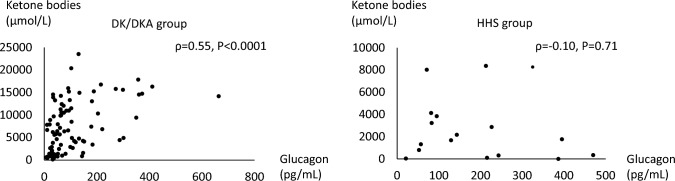
Associations of plasma glucagon levels with serum ketone bodies in the DK/DKA group and the HHS group. *DK* diabetic ketosis, *DKA* diabetic ketoacidosis, *HHS* hyperosmolar hyperglycemic state

A positive correlation was seen between the plasma glucagon level and the serum ketone bodies level in the DK/DKA group, but no correlation was seen in the HHS group (Fig. 1).

### Factors associated with plasma glucagon levels in type 1 and type 2 diabetes mellitus in the DK/DKA group and the HHS group

**Fig. 2:**
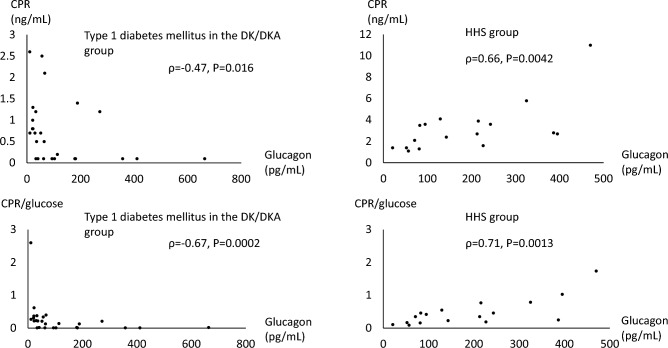
Associations of plasma glucagon levels with serum C-peptide immunoreactivity (CPR) (upper panels) and serum CPR/glucose ratio (lower panels) in type 1 diabetes mellitus in the DK/DKA group and the HHS group. Serum CPR/glucose ratio is shown as calculated values multiplied by 100. *DK* diabetic ketosis, *DKA* diabetic ketoacidosis, *HHS* hyperosmolar hyperglycemic state

In patients with type 1 diabetes mellitus in the DK/DKA group (Table 3, Fig. 2), the plasma glucagon level was positively correlated with plasma glucose, serum osmolality, and serum creatinine, whereas a negative correlation was seen with serum CPR (ρ = − 0.47, *P* = 0.016) and serum CPR/plasma glucose ratio (ρ = − 0.67, *P* = 0.0002). In patients with type 2 diabetes mellitus in the DK/DKA group (Table 3), the plasma glucagon level was positively correlated with plasma glucose, serum osmolality, and serum creatinine, and also positively with serum CPR (ρ = 0.25, *P* = 0.0429). No correlation was seen with serum CPR/plasma glucose ratio (ρ = − 0.18, *P* = 0.14).

**Table 3:** Factors associated with plasma glucagon levels in type 1 and type 2 diabetes mellitus in the DK/DKA group, and the HHS group

	*Ρ*	*P* value
Type 1 diabetes mellitus in the DK/DKA group (N = 26)		
Glucose	0.68	0.0001
Serum CPR	− 0.47	0.016
CPR/glucose	− 0.67	0.0002
Serum OSM	0.72	< 0.0001
Creatinine	0.65	0.0003
Type 2 diabetes mellitus in the DK/DKA group (N = 66)		
Glucose	0.60	< 0.0001
Serum CPR	0.25	0.043
CPR/glucose	− 0.18	0.14
Serum OSM	0.61	< 0.0001
Creatinine	0.66	< 0.0001
HHS group (N = 17)		
Glucose	− 0.44	0.076
Serum CPR	0.66	0.0042
CPR/glucose	0.71	0.0013
Serum OSM	0.41	0.10
Creatinine	0.44	0.080

*DK* diabetic ketosis, *DKA* diabetic ketoacidosis, *HHS* hyperosmolar hyperglycemic state, *CPR* C-peptide immunoreactivity, *OSM* osmolality

In the HHS group, no correlations were seen with plasma glucose, serum osmolality, or serum creatinine levels, and a positive correlation was seen with serum CPR (ρ = 0.66, *P* = 0.0042) and serum CPR/plasma glucose ratio (ρ = 0.71, *P* = 0.0013) (Table 3, Fig. 2).

### Relationships among duration of hyperglycemic symptoms and duration of diabetes, and plasma glucagon and serum CPR levels in patients with type 1 and type 2 diabetes mellitus in the DK/DKA group

**Fig. 3:**
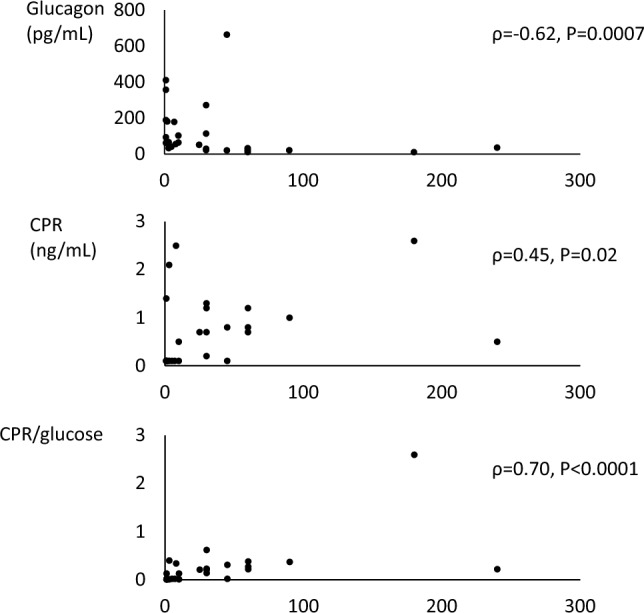
Associations of duration of hyperglycemic symptoms with plasma glucagon levels (upper panel), serum C-peptide immunoreactivity (CPR) (middle panel) and serum CPR/glucose ratio (lower panel) in type 1 diabetes mellitus in the DK/DKA group. X axis denotes duration of hyperglycemic symptoms (days). Serum CPR/glucose ratio is shown as calculated values multiplied by 100

In patients with type 1 diabetes mellitus (Fig. 3), duration of hyperglycemic symptoms was negatively correlated with plasma glucagon levels (ρ = − 0.62, *P* = 0.0007). No correlation was seen with duration of diabetes (ρ = 0.23, *P* = 0.26). Duration of hyperglycemic symptoms was positively correlated with serum CPR (ρ = 0.45, *P* = 0.02) and serum CPR/plasma glucose ratio (ρ = 0.70, *P* < 0.0001). In patients with type 2 diabetes mellitus, no correlation was seen between duration of hyperglycemic symptoms and duration of diabetes, and plasma glucagon and serum CPR levels (data not shown).

### Pre- and post-treatment plasma glucagon levels in the DK/DKA group and the HHS group

**Fig. 4:**
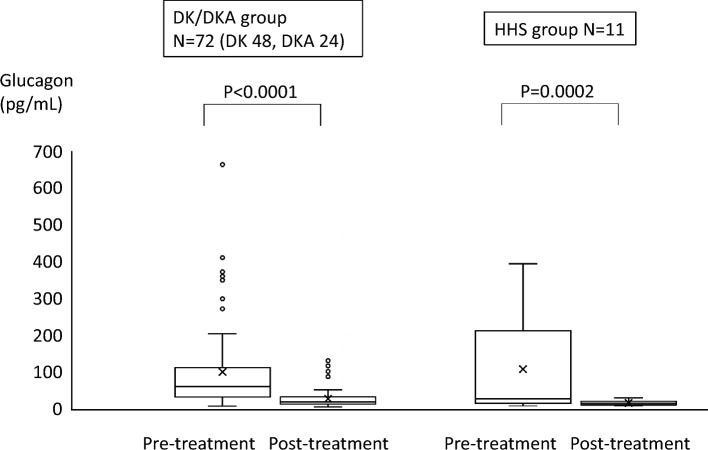
Pre- and post-treatment plasma glucagon levels in the DK/DKA group and the HHS group. *DK* diabetic ketosis, *DKA* diabetic ketoacidosis, *HHS* hyperosmolar hyperglycemic state

In both the DK/DKA and HHS groups, the plasma glucagon level was significantly decreased from before to after treatment (Fig. 4). Median plasma glucagon levels decreased from 61.1 to 19.1 pg/mL in the DK/DKA group and from 212.7 to 16.9 pg/mL in the HHS group. The plasma glucagon level after treatment was within the normal reference ranges in a majority of patients.

## Discussion

In this study, hyperglucagonemia, despite hyperglycemia, was seen during DK/DKA and HHS using measurements of pancreas-specific glucagon with a sandwich ELISA. Plasma glucagon levels were higher in the HHS group than in the DK/DKA group. In the DK/DKA group, hyperglucagonemia and the serum ketone bodies level were positively correlated, but that correlation was not seen in the HHS group. In patients with type 1 diabetes mellitus in the DK/DKA group, hyperglucagonemia was correlated with a decrease in insulin secretion relative to hyperglycemia. In the HHS group, hyperglucagonemia was positively correlated with insulin secretion. As a major finding of this study, the possibility was suggested that the cause of hyperglucagonemia differs in DK/DKA due to type 1 diabetes mellitus and HHS due to type 2 diabetes mellitus. Plasma glucagon levels decreased to within the normal reference ranges in a majority of patients, further supporting the presence of hyperglucagonemia in DK/DKA and HHS.

The plasma glucagon level was higher in the HHS group than in the DK/DKA group. This result agrees with previous reports using RIA [14, 15]. One mechanism for this is thought to be that, in the HHS group, serum osmolality and the creatinine level are high, and dehydration is severe. The kidneys are known to play an important role in the clearance of glucagon, and in previous reports as well, renal dysfunction was shown to be correlated with elevated plasma glucagon levels [20].

In the DK/DKA group, a positive correlation was seen between plasma glucagon levels and serum ketone bodies levels. In the pathogenesis of DK/DKA, the glucagon/insulin ratio increases with decreased insulin secretion. This causes the acceleration of gluconeogenesis and glycogen breakdown in the liver and decreased glucose disposal in peripheral tissue, leading to hyperglycemia. At the same time, free fatty acids are released from adipose tissue with lipolysis, causing ketone bodies production in the liver [21]. Because of this, ketone bodies production reflects the severity of DK/DKA. Glucagon is a major hormone involved in ketone bodies production [22]. It is reasonable that a correlation between the plasma glucagon level and the serum ketone bodies level was seen in the DK/DKA group. With HHS, however, hypovolemia from osmotic diuresis due to hyperglycemia is severe, but as shown in the present study, insulin secretion remains preserved to some extent, and lipolysis and ketone bodies production is limited [22, 23]. This is thought to be why no correlation was seen between plasma glucagon and serum ketone bodies levels in the HHS group.

In the correlation between plasma glucagon levels and clinical factors, a positive correlation was seen with plasma glucose, serum osmolality and creatinine in both type 1 and type 2 diabetes patients in the DK/DKA group, a result reflecting hypovolemia and disease severity. In patients with type 1 diabetes mellitus in the DK/DKA group, the plasma glucagon level was negatively correlated with serum CPR and serum CPR/plasma glucose ratio, suggesting relationships with decreased insulin secretion and hyperglucagonemia. In contrast, in the HHS group with all type 2 diabetes patients, the plasma glucagon level was positively correlated with serum CPR and serum CPR/plasma glucose ratio. As mentioned in the Introduction, insulin suppresses glucagon secretion. There is a report that the cause of hyperglucagonemia differs between type 1 and type 2 diabetes mellitus [24]. In that report, decreased insulin secretion in type 1 diabetes mellitus, but exaggerated insulin response in type 2 diabetes mellitus, was correlated with the exaggerated glucagon response to the arginine challenge test. In type 2 diabetes mellitus, positive correlations were also seen between both an exaggerated glucagon response and an estimate of insulin resistance. From the above, insulin deficiency was considered to be a cause of hyperglucagonemia in type 1 diabetes mellitus, whereas in type 2 diabetes mellitus, in addition to systemic insulin resistance, insulin resistance in α cells may be a cause of hyperglucagonemia [24]. Similar to this previous report, the possibility is suggested that decreased insulin secretion in DK/DKA with type 1 diabetes mellitus and possible α cell insulin resistance in HHS with type 2 diabetes mellitus are causes of hyperglucagonemia. Even in patients with type 2 diabetes mellitus in the DK/DKA group, a positive correlation was seen between plasma glucagon and serum CPR levels, similar to HHS, and the possibility was suggested that α cell insulin resistance is a cause of hyperglucagonemia.

In patients with type 1 diabetes mellitus in the DK/DKA group, shorter duration of hyperglycemic symptoms was associated with hyperglucagonemia and also with lower endogenous insulin secretion. These findings are consistent with the negative correlation between plasma glucagon level and decreased insulin secretion. In patients with type 2 diabetes mellitus, such associations were not observed, possibly due to the difficulties to determine duration of hyperglycemic symptoms.

After treatment, the plasma glucagon level normalized in a short time after infusion and insulin administration in the majority of patients in both the DK/DKA group and the HHS group. Previous reports that hyperglucagonemia in DK/DKA normalizes with infusion and insulin administration agree with the results of the present study [14, 22]. In HHS, meanwhile, it has also been reported that hyperglucagonemia normalizes with improvement of hypovolemia and normalization of hemodynamics [25]. In the present study, the plasma glucagon level normalized after the administration of insulin in both groups, and correction of insulin action is thought to contribute to improvement in hyperglucagonemia. However, since multiple treatments including treatment for dehydration are done simultaneously with the administration of insulin, it is unclear which factors were mostly effective to decrease the plasma glucagon level.

The limitations in this study are, first, the small numbers of type 1 diabetes patients (n = 26) and HHS patients (n = 17). Multiple regression analysis of the factors contributing to the plasma glucagon level could not be performed in either group. Moreover, though no correlation was seen between the plasma glucagon level and serum osmolality or creatinine level in HHS patients, in a scatter plot of these correlations, extreme outliers were seen (data not shown) and are thought to have affected the statistical analysis. The number of patients needs to be increased. Second, this was a retrospective study, and so although the timing of laboratory tests related to the hyperglycemic crisis and the timing of treatments including insulin and infusion were confirmed from the medical records, the possibility that errors occurred in some patients cannot be completely ruled out. Finally, with the sandwich ELISA used in the present study, cross-reactivity with glicentin is reported to affect the measurement results in some patients [26, 27]. There are no reports of glicentin secretion in hyperglycemic crises, and the effect is unclear. New sandwich ELISA methods to minimize the effects of proglucagon-derived peptides are being developed [28]. In the future, it will be necessary to conduct assessments with these new ELISA methods.

In conclusion, with measurements of pancreas-specific glucagon by a sandwich ELISA, this study showed that hyperglucagonemia exists in DK/DKA and HHS. In DK/DKA, a relationship between hyperglucagonemia and ketogenesis was shown. The possibility was suggested that the cause of hyperglucagonemia differs in DK/DKA due to type 1 diabetes mellitus and in HHS due to type 2 diabetes mellitus. The plasma glucagon level normalized within a short time after infusion and insulin administration. In further studies, analysis of the relationships of the decrease in plasma glucose and serum ketone bodies levels, and improvement in dehydration with the decrease in plasma glucagon level over the course of treatment would be beneficial in formulating more appropriate treatment plans for DK/DKA and HHS.

## Supplementary Information

Below is the link to the electronic supplementary material.

Supplementary Figure. Associations of serum ketone bodies levels with pH, anion gap (AG) and bicarbonate in arterial blood in the DK/DKA group. Open circles; DK and filled circles; DKA. X axis denotes serum ketone bodies (μmol/L). The analysis was performed using Spearman‘s rank correlation coefficient. Note that substantial overlap is seen between DK and DKA in the associations of serum ketone bodies levels with AG and bicarbonate in arterial blood. (PPTX 309 KB)
