# Supplementary material for: Differential association of hyperglucagonemia with C-peptide levels in diabetic ketosis/ketoacidosis and hyperosmolar hyperglycemic state
Source: Diabetol Int. 2025 Nov 23;17(1):1. doi: 10.1007/s13340-025-00852-8 (PMC12640887; doi:10.1007/s13340-025-00852-8)
Supplement: Supplementary file 1 — Supplementary Figure. Associations of serum ketone bodies levels with pH, anion gap (AG) and bicarbonate in arterial blood in the DK/DKA group. Open circles; DK and filled circles; DKA. X axis denotes serum ketone bodies (μmol/L). The analysis was performed using Spearman‘s rank correlation coefficient. Note that substantial overlap is seen between DK and DKA in the associations of serum ketone bodies levels with AG and bicarbonate in arterial blood. (PPTX 309 KB) [file 13340_2025_852_MOESM1_ESM.pptx]

## Slide 1
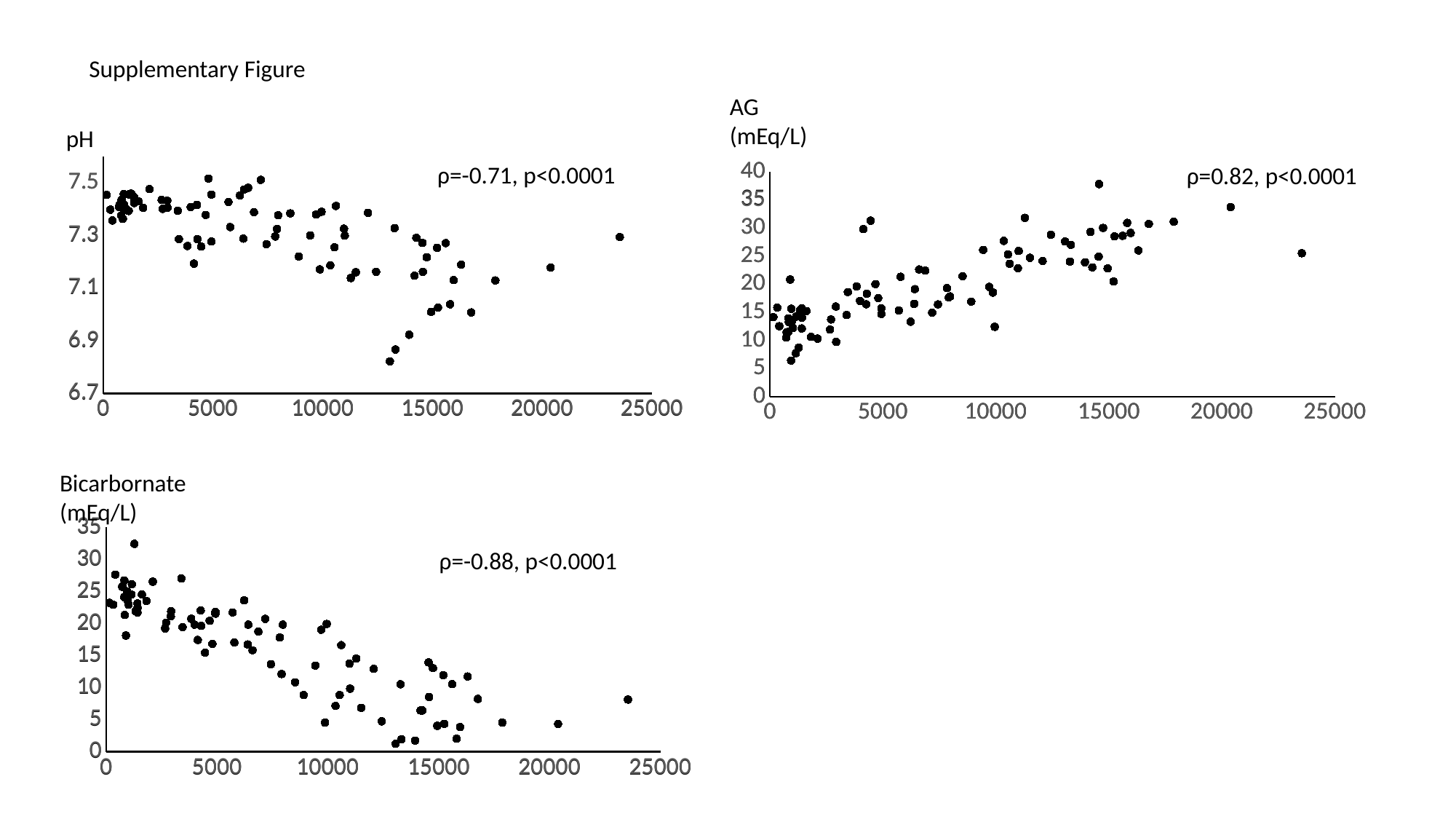

Supplementary Figure
AG
(mEq/L)
pH
### Chart
| Category | ｐH |
|---|---|
### Chart
| Category | ｐH |
|---|---|
### Chart
| Category | ｐH |
|---|---|
### Chart
| Category | ｐH |
|---|---|ρ=-0.71, p<0.0001
ρ=0.82, p<0.0001
Bicarbornate
(mEq/L)
### Chart
| Category | ｐH |
|---|---|
### Chart
| Category | ｐH |
|---|---|ρ=-0.88, p<0.0001
